# Supplementary material for: Identification of photoperiod-induced specific miRNAs in the adrenal glands of Sunite sheep (Ovis aries)
Source: Front Vet Sci. 2022 Jul 22;9:888207. doi: 10.3389/fvets.2022.888207 (PMC9354845; doi:10.3389/fvets.2022.888207)
Supplement: Supplementary file 1 [file Data_Sheet_1.ZIP › Supplementary file/miRDeep2 file.zip!/miRDeep2 file/Know_Structure/SPLP42A2/oar-mir-199a.pdf]

miRBase precursor : oar-mir-199a
Total read count : 134435
oar-miR-199a-3p read count : 88511
remaining reads : 45924

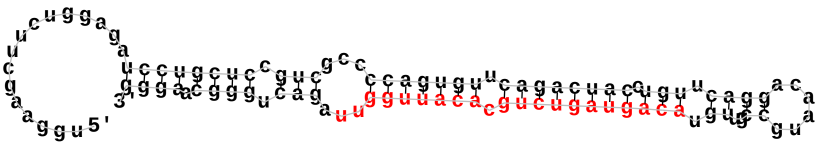

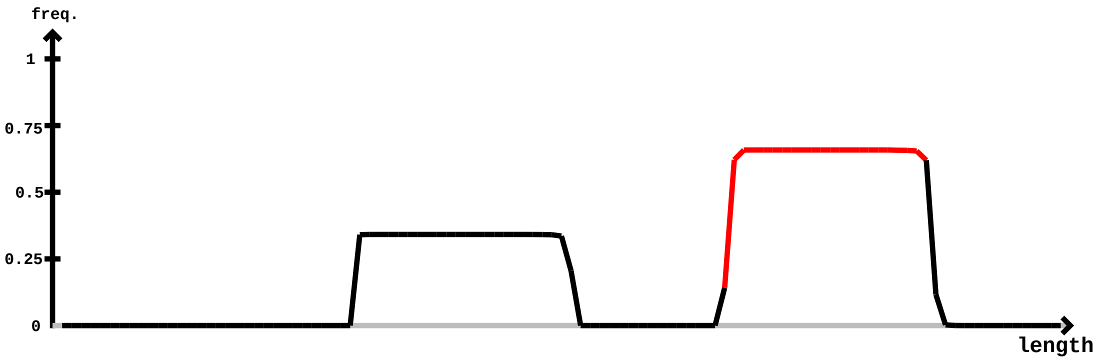


```
                                                                                          oar-miR-199a-3p
5'- uggaagcuucuggagauccugcuccgucgccccaguguucagacuaccuguucaggacaaugccguuguacaguagucugcacauugguuagacugggcaaggg   -3'   exp
    .................(((((((.(((...(((((((.((((((((.(((.((((......))..)).))))))))))).)))))))...))).)))).))).   reads    mm    sample
    .............................ccccaguguucagacuaccuguu....................................................   8        0     seq
    .............................ccccaguguucagacuaccuguuc...................................................   1        0     seq
    ..............................cccaguguucagacuac.........................................................   1        0     seq
    ..............................cccaguguucagacuacc........................................................   7        0     seq
    ..............................cccaguguucagacuaccu.......................................................   36       0     seq
    ..............................cccaguguucagacuaccug......................................................   88       0     seq
    ..............................cccaguguucagacuaccugu.....................................................   677      0     seq
    ..............................cccaguguucagacuaccuguu....................................................   17425    0     seq
    ..............................cccaguguucagacuaccuguuc...................................................   27574    0     seq
    ...............................ccaguguucagacuaccugu.....................................................   2        0     seq
    ...............................ccaguguucagacuaccuguu....................................................   27       0     seq
    ...............................ccaguguucagacuaccuguuc...................................................   72       0     seq
    ................................caguguucagacuaccuguu....................................................   2        0     seq
    ................................caguguucagacuaccuguuc...................................................   1        0     seq
    ....................................................caggacaaugccguuguacagu..............................   1        0     seq
    .....................................................aggacaaugccguuguacaguagu...........................   1        0     seq
    ...........................................................augccguuguacaguagucu.........................   1        0     seq
    ....................................................................uacaguagucugcacau...................   1        0     seq
    ....................................................................uacaguagucugcacauu..................   38       0     seq
    ....................................................................uacaguagucugcacauug.................   18       0     seq
    ....................................................................uacaguagucugcacauugg................   158      0     seq
    ....................................................................uacaguagucugcacauuggu...............   2165     0     seq
    ....................................................................uacaguagucugcacauugguu..............   15611    0     seq
    ....................................................................uacaguagucugcacauugguua.............   1047     0     seq
    ....................................................................uacaguagucugcacauugguuag............   14       0     seq
    .....................................................................acagCagucugcacauu..................   1        1     seq
    .....................................................................acaguagucugcacauu..................   93       0     seq
    .....................................................................acaAuagucugcacauu..................   2        1     seq
    .....................................................................acaguagucugcacauug.................   30       0     seq
    .....................................................................acaguagucugcacauugg................   170      0     seq
    .....................................................................acaguagucugcacauuggu...............   2282     0     seq
    .....................................................................acaguagucugcacauugguu..............   48429    0     seq
    .....................................................................acaguagucugcacauugguua.............   13203    0     seq
    .....................................................................acaguagucugcacauugguuag............   273      0     seq
    .....................................................................acaguagucugcacauugguuaga...........   16       0     seq
```

oar-miR-199a-3p

```
uggaagcuucuggagauccugcuccgucgccccaguguucagacuaccuguucaggacaaugccguuguacaguagucugcacauugguuagacugggcaaggg
.....................................................................acaguagucugcacauugguuagG...........   4      1      seq
......................................................................caguagucugcacauug.................   2      0      seq
......................................................................caguagucugcacauugg................   10     0      seq
......................................................................caguagucugcacauuggu...............   221    0      seq
......................................................................caguagucugcacauugguu..............   3625   0      seq
......................................................................caguagucugcacauugguua.............   1026   0      seq
......................................................................caguagucugcacauugguuag............   34     0      seq
......................................................................caguagucugcacauugguuagG...........   1      1      seq
......................................................................caguagucugcacauugguuaga...........   4      0      seq
.......................................................................aguagucugcacauuggu...............   1      0      seq
.......................................................................aguagucugcacauugguu..............   20     0      seq
.......................................................................aguagucugcacauugguua.............   10     0      seq
.......................................................................aguagucugcacauugguuag............   1      0      seq
........................................................................guagucugcacauugguu..............   1      0      seq
```
